# Supplementary material for: Could the Human Endogenous Retrovirus-Derived Syncytialization Inhibitor, Suppressyn, Limit Heterotypic Cell Fusion Events in the Decidua?
Source: Int J Mol Sci. 2021 Sep 23;22(19):10259. doi: 10.3390/ijms221910259 (PMC8508417; doi:10.3390/ijms221910259)
Supplement: Supplementary file 1 [file ijms-22-10259-s001.zip › Supplementary Figures_revised.pdf]

Supplementary Figures

Could the Human Endogenous Retrovirus-Derived Syncytialization Inhibitor, Suppressyn, Limit Heterotypic Cell Fusion Events in the Decidua?

Jun Sugimoto [1], Sehee Choi [2], Megan A. Sheridan [2], Iemasa Koh [1], Yoshiki Kudo [1] and Danny J. Schust [2,*]

1. Department of Obstetrics and Gynecology, Graduate School of Biomedical & Health Science, Hiroshima University, 1-2-3 Kasumi, Minami-ku, Hiroshima 734-8551, Japan; juns@hiroshima-u.ac.jp (J.S.); iemasako@hiroshima-u.ac.jp (I.K.); yoshkudo@hiroshima-u.ac.jp (Y.K.)
2. Department of Obstetrics, Gynecology and Women's Health, Division of Reproductive Endocrinology and Infertility, University of Missouri, 500 North Keene Street, Suite 203, Columbia, MO 65201, USA; seheechoi@missouri.edu (S.C.); mahrmf@health.missouri.edu (M.A.S.)

* Correspondence: schustd@health.missouri.edu (D.J.S.)

Supplementary figure 1

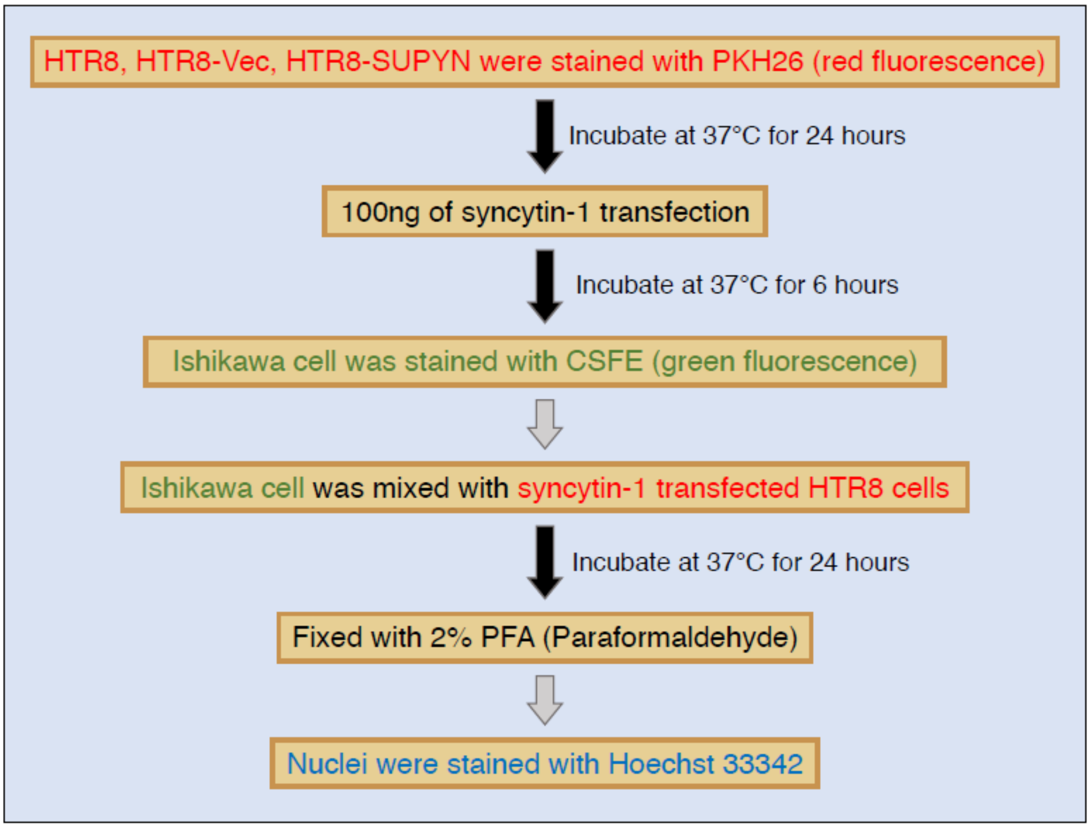

Figure S1. Outline of Experimental Methods.

Supplementary figure 2

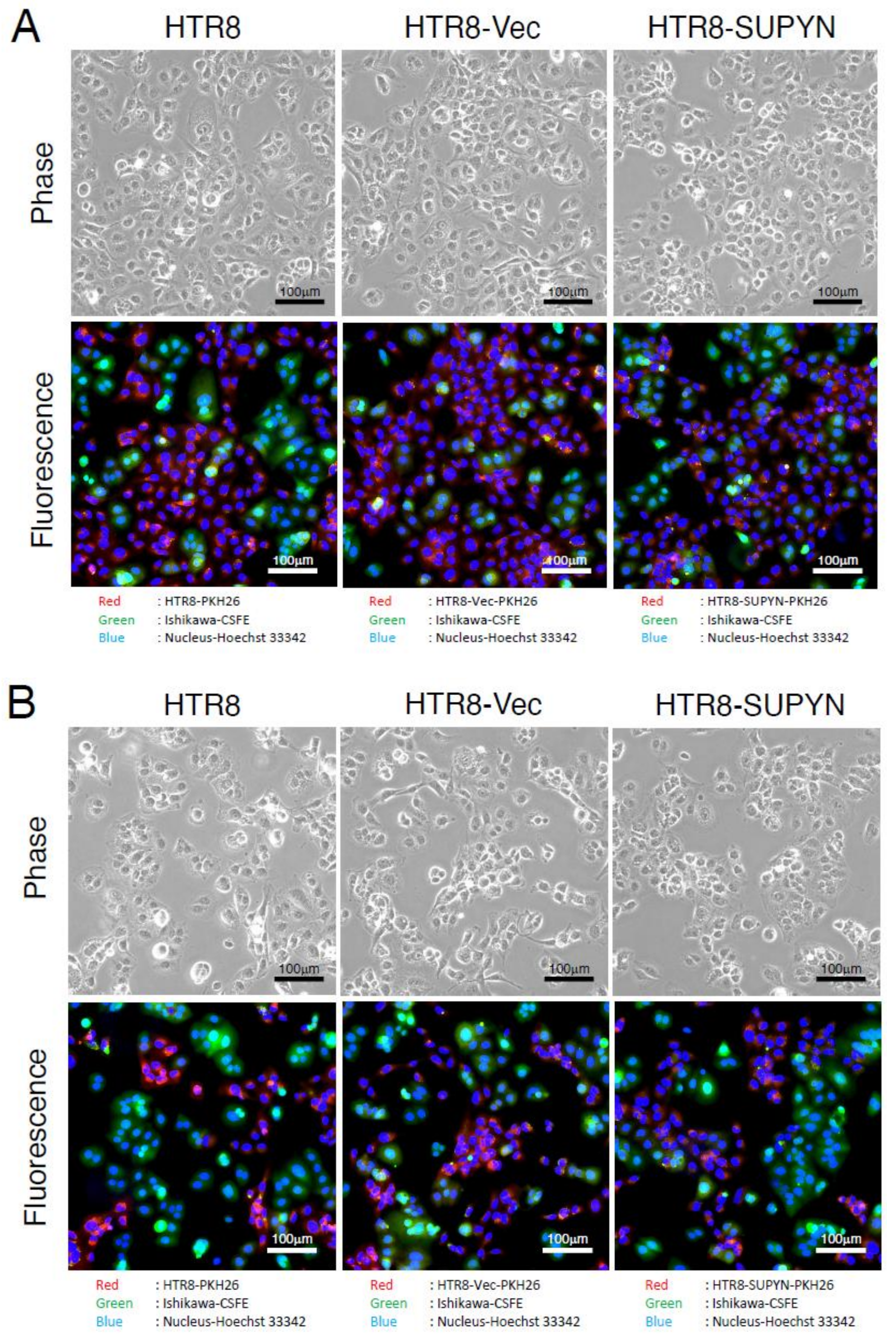

Figure S2. Control experiments for HTR8-Ishikawa cell co-cultures. HTR8 trophoblast cells (HTR8) and its control and ERVE48-1transfected derivatives, were labeled with a red dye. Ishikawa endometrial cells were labeled with a green dye and placed in co-culture with HTR8, HTR8 transfectants and their controls to assess fusion. These experiments are depicted in Figure 3 in the main manuscript. HTR8 (red) were treated lipofectamine 2000 only without additional plasmid vectors (A) or with the syn1 control vector (B) prior to co-culture with Ishikawa endometrial cells (green). All cell nuclei were counterstained blue with Hoechst 33342. Phase contrast images are presented for comparison to aid in distinguishing cell boundaries.

Supplementary figure 3

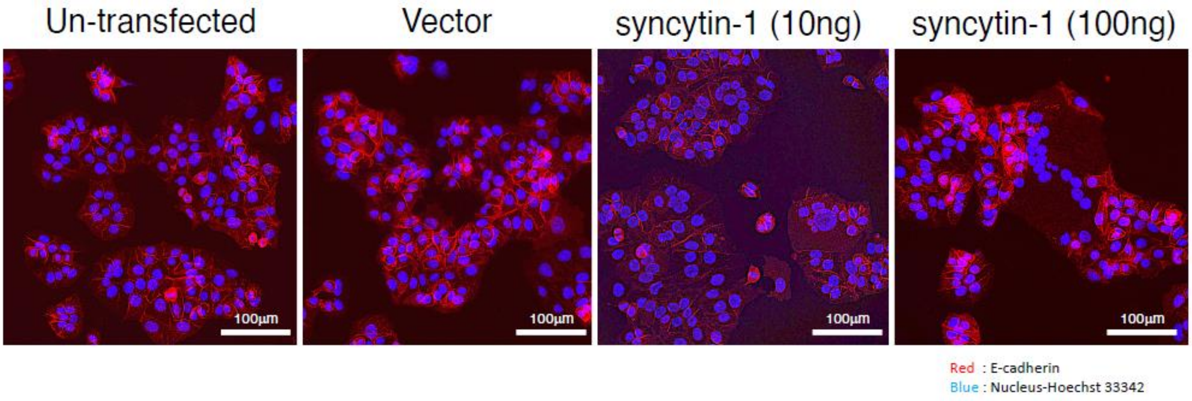

Figure S3. Cell fusion of Ishikawa cells upon transient transfection of syn1. Ishikawa cells were transiently transfected with syn1 (10 ng or 100 ng) and cultured for 24 h. They were then fixed and stained with an anti-E-cadherin antibody (# 3195: CST Danvers, MA, USA: diluted 1/500) followed by an anti-rabbit-IgG-Alexa fluor 555 secondary antibody (A21429: Thermo Fisher Scientific, Waltham, MA, USA). Cells were counterstained with 0.5 µg/mL of Hoechst 33342 (H342 Dojindo, Kumamoto, Japan) for nuclear staining. Fluorescent signals were detected using a fluorescence microscope (BZ-X710; KEYENCE Japan, Osaka, Japan).
